# Understanding retinoblastoma: epidemiology and genetics

**Published:** 2018-06-03

**Authors:** Ido Didi Fabian, Mandeep S Sagoo

**Affiliations:** 1 Consultant Ocular Oncologist: Ocular Oncology Service, Goldschleger Eye Institute, Sheba Medical Center, Tel Aviv, Israel.; 2 Retinoblastoma Service: Royal London Hospital; Ocular Oncology Service NIHR Biomedical Research Centre for Ophthalmology, Moorfields Eye Hospital and UCL Institute of Ophthalmology, London UK.

**Retinoblastoma is usually initiated by a random mutation of a gene in a retinal cell. It is important to try and recognise if the child has germline retinoblastoma, as this may affect both eyes of the child. Siblings and future children of the child with retinoblastoma are at greater risk of developing this cancer.**

**Figure 1:**
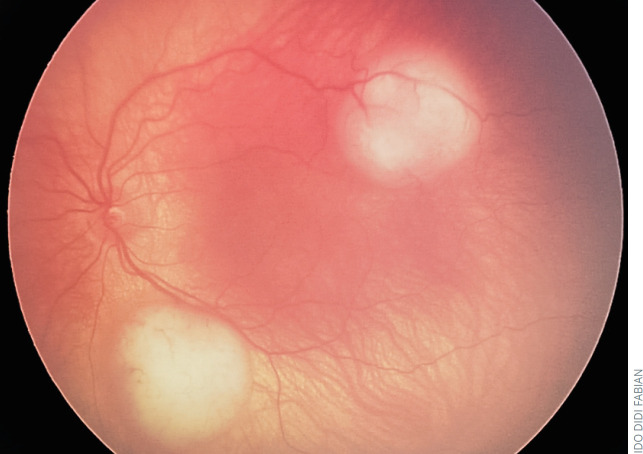
Multifocal retinoblastoma, suggesting germline disease

Retinoblastoma is the most common eye cancer of childhood. However, it is a relatively rare disease, occurring in approximately one out of every 16,000–18,000 live births in the global population.^1^ Its incidence is similar across populations, and does not vary according to gender, ethnicity or socio-economic status. Worldwide, approximately 8,000 children develop retinoblastoma each year, with the vast majority presenting with the disease before the age of 5 years.

Retinoblastoma originates in a photoreceptor cell of the retina and is associated with a mutation in the *RB1* gene (which is normally responsible for tumour suppression). Each person inherits two copies (or alleles) of the *RB1* gene - one from each parent. For retinoblastoma to occur, both copies in a single retinal cell must undergo a mutation.

Retinoblastoma is categorised by whether the mutation is germline (hereditary) or non-germline (occuring sporadically).

## Germline retinoblastoma

In many cases of germline retinoblastoma, a mutated *RB1* allele is inherited from a parent and is present in all the cells of a child's body. A second mutation in a retinal cell results in a tumour.

Children with germline retinoblastoma often present at a young age (median of 15 months).

Tumours can occur in multiple locations in one eye (multifocal disease, Figure 1) and in both eyes (bilateral).

A child that presents with bilateral disease is 100% likely to have a germline mutation. Germline retinoblastoma may occur in an asymmetrical manner, with a different grade of tumour in each eye at presentation. Children may even present with unilateral retinoblastoma initially, becoming bilateral later.

It is very important that germline retinoblastoma is recognised. If you see a child with retinoblastoma in one eye, do not assume that it is non-germline: it is estimated that 10–20% of children who present with unilateral disease have germline retinoblastoma. Ask about family history of retinoblastoma, or about removal of an eye in childhood of a family member, and **always examine the second eye to look for early signs of tumour.** Refer the family for genetic counselling and testing, if available (see p. 8).

### Retinoma

A retinoma is a benign precursor of retinoblastoma. Retinomas should be looked for in the parents of an affected child. The presence of a retinoma in a parent or sibling confirms that the disease is inherited (and therefore germline) in that family.

### Mosaicism

Occasionally, a random mutation in the *RB1* gene may occur very soon after conception. Depending on the stage of development at which this occurs, some of the child's body cells will have the mutated *RB1* gene, and others will not; this is known as mosaicism. If the mutated *RB1* gene is present in the germ cells, the child can pass the mutation on to future generations.

## Non-germline retinoblastoma

Non-germline retinoblastoma affects just one eye. It is the more common type of retinoblastoma. As the name implies, non-germline retinoblastoma is not inherited from parents and cannot be passed on to future generations. It develops due to two random mutations in the *RB1* gene in one cell of the retina. It is also known as ‘sporadic’ or ‘somatic’ retinoblastoma.

Recently, it has been found that retinoblastoma may occur even in the presence of non-mutated *RB1* genes, due to activity associated with the MYCN oncogene.^2^ This is rare, occurring in fewer than 3% of children with unilateral retinoblastoma. It tends to present at an earlier age, often before 6 months.
